# Bronchus-associated lymphoid tissue lymphoma (BALT) in a patient with primary Sjögren’s syndrome

**DOI:** 10.31138/mjr.28.1.52

**Published:** 2017-03-28

**Authors:** Evangelia N. Mole, Vasilios T. Papadakos, Charalampos I. Sfontouris

**Affiliations:** Department of Rheumatology, Evaggelismos General Hospital, Athens, Greece

**Keywords:** Sjögren’s syndrome, BALT lymphoma, R-CHOP, biopsy

## Abstract

Bronchus-associated lymphoid tissue lymphoma (BALT) is classified as marginal-zone lymphoma. Although it is a rare disease, the possibility of development in patients with Sjögren’s syndrome should always be included in the physician’s mind. Usually it appears as a distinct clinical and radiological entity. We describe a case of BALT lymphoma in a patient with primary Sjögren’s syndrome (pSS) who presented with a solitary mass and several smaller focal infiltrations in the right lower lobe of the lung. The definitive diagnosis was achieved after performing an open biopsy and histological examination of the lesion. It is considerably difficult to identify lymphoma by computed tomography exclusively, since there is a great discrepancy between clinical and radiological features. A lesion biopsy should always be performed.

## INTRODUCTION

Sjögren’s syndrome (SS) is a systemic autoimmune disease of the exocrine glands, formally associated with focal and progressive lymphoplasmacytic infiltration, resulting in dry mouth and eye. Patients with SS are also known to have an increased risk of developing B-cell non-Hodgkin’s lymphoma (BNHL), mainly in the salivary glands and other mucosa-associated lymphoid tissues (MALT). We quote a case of bronchus-associated lymphoid tissue lymphoma (BALT) with bone marrow invasion and B-cell symptomatology in a patient with pSS, who was successfully treated with surgery and chemotherapy.

## CASE DESCRIPTION

A 63-year-old Caucasian woman with a history of pSS was admitted to our clinic for investigation of palpable purpura of lower limbs and generalized weakness persisted over the last 4 months. She reported having dry cough, dry skin and intense pruritus for 6 months. Recent laboratory findings (2 months ago) included leucopenia, elevated inflammation markers and γ-globulins, positive rheumatoid factor and antinuclear antibodies. A computed tomography (CT) scan of the chest revealed nodular density in the lower right lobe measuring 2×3cm. Furthermore, the patient had a history of insulin-treated diabetes mellitus, arterial hypertension (receiving felodipine, carvedilol, ramipril and furosemide), hypothyroidism and osteoporosis (receiving alendronate sodium). The diagnosis of pSS was made 22 years ago, based on arthritis, intense sicca features, positive antinuclear antibodies (ANA), anti-extractable nuclear antigens antibodies (ENA) (anti-Ro, anti-La Abs), rheumatoid factor (RF) and biopsy of minor salivary gland indicative of pSS (Tarpley score 3).

**Figure 1:**
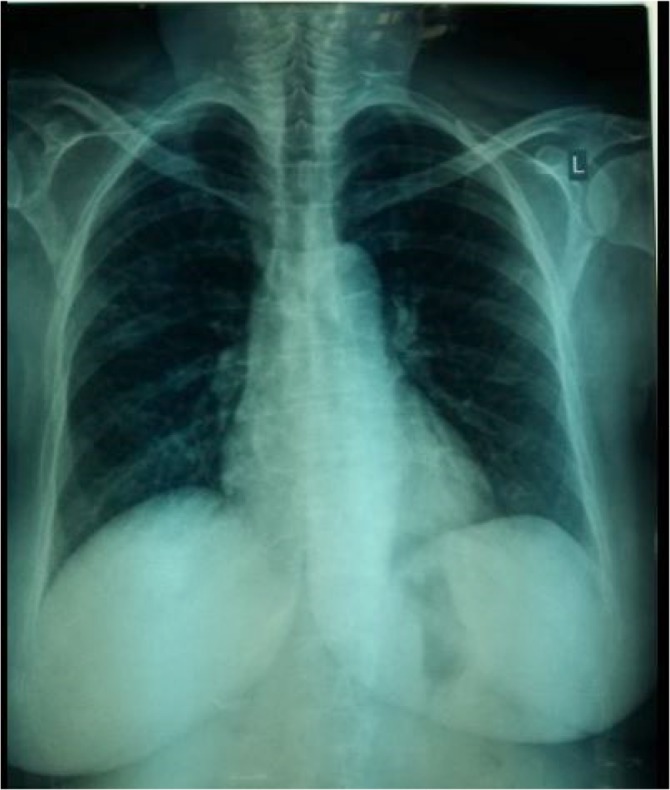
Chest radiograph on admission

**Figure 2:**
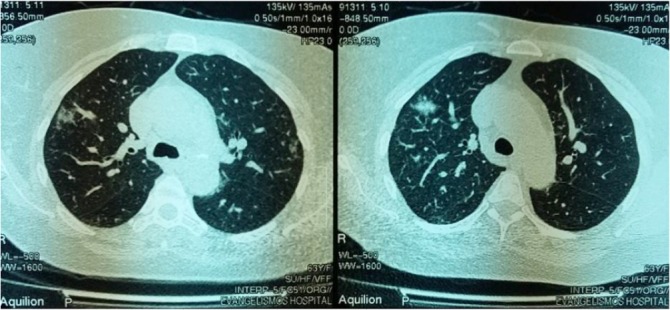
Chest CT scan showing a compact spindle – shaped mass in the right lower lobe surrounded by focal infiltration

During the clinical evaluation, the patient looked normal without any acute distress; also, her vital signs were within normal range. Examination revealed enlarged, painless and moveable submandibular glands and palpable cervical lymph nodes. Chest examination revealed crackles of the right lung. Arthritis of the left ankle and palpable purpura of lower limbs were also observed. The rest of the physical examination, regarding cardiovascular, gastrointestinal and central nervous system, was normal. Laboratory data (**Table 1**) showed anemia, leucopenia, lymphocytopenia, elevated levels of ESR, CRP and β2-microglobulin, positive ANA and ENA (Ro, La), negative RF, anti-neutrophil cytoplasmic antibodies (ANCA Abs) and cryoglobulins, elevated γ–globulins region in a diffuse pattern and normal values of complement. The chest x-ray was normal (**Figure 1**). The cervical ultrasound confirmed the enlarged lymph nodes (the biggest was 1.3cm in diameter). A computed tomography (CT) scan of the chest was repeated and it revealed a compact spindle–shaped mass in the right lower lobe measuring 2cm in diameter, accompanied by focal infiltration around the mass and several smaller focal infiltrations in the right lobe (**Figure 2**). The CT scan of the abdomen showed enlarged para-aortic lymph nodes in the kidneys. Spirometry was normal and bronchoscopy failed to detect visible intrabronchial lesion. Cultures of biologic fluids were negative. Both bronchoalveolar lavage cultures and cytology were negative for infection and malignancy respectively.

**Table 1:** Laboratory data on admission

		Normal values
Hct/Hbg	34.8/11.2	37–47%/12–15gr/dl
MCV/MCH	83.7/27.6	80–98fl/27–33pg/cell
WBC	3650	4000–10500
Neu/Lym	2160/1040	2000–7700/1500–4000/μl
PLT	152000	140–450×10^3^/μl
ESR	115	<20mm/h
CRP	2	0.5mg/dl
LDH	171	<225 IU/L
RF	19.7	<20 IU/ml
ANA	1/640, speck-led pattern	
Anti ds-DNA	Negative	
ENA (Ro, La)	Positive	
ANCA C, P	Negative	
IgG/IgA/IgM	3050/715/133	690–1618/72–400/40–235mg/dl
C3, C4	80.4/14	63–158/14–33mg/dl
β2-microglobulin	3667.5	<1900μg/lt
Cryoglobulines	negative	

According to the past history of the patient, the clinical examination and laboratory findings, the following were considered in the differential diagnosis: underlying lymphoproliferative disorder, bronchioalveolar carcinoma, sarcoidosis, lymphocytic interstitial pneumonitis and non-specific interstitial pneumonia. Infection was considered unlikely, since she did not have fever, purulent sputum and cultures of biologic fluids, and purified protein derivative (PPD) skin test were negative.

Patient underwent right limited thoracotomy by thoracoscope and informal excision of lung parenchyma and mass was performed. The biopsy showed involvement of lung parenchyma by a “lymphoid proliferation” and formation of lymphoid nodules, comprised mainly of lymphocytes and plasma cells. The lymphoid infiltration involved interstitial tissue and alveoli. The rest parenchyma showed limited findings of lymphocytic interstitial pneumonitis (LIP). On immunostain, the lymphocyte population intensely expressed CD20 antigen, but was negative for CD5, CD23 antigens and cyclin D1 protein. The lymphocytic cells also showed kappa Ig light chain restriction. Findings in favor of B-non-Hodgkin lymphoma, type of mucosa-associated lymphoid tissue (MALT) with intense plasmacytic differentiation. Furthermore, bone marrow was infiltrated by small lymphocytes. On immunostain, lymphocytes expressed CD3 and CD20 antigens in overall percentage 10%.

Based on the clinical presentation, radiologic appearance and histologic examination, the patient was diagnosed with pulmonary marginal zone B-cell lymphoma of bronchus-associated lymphoid tissue with plasmacytic differentiation, bone marrow invasion and presence of B-cell symptoms. She was categorized as BALT lymphoma stage IV B, according to the Ann Arbor staging system. She received 6 cycles of R-CHOP (Rituximab, cyclophosphamide, doxorubicin, vincristine and prednisolone) and continues to do well 20 months after the diagnosis, with no signs of lymphoma recurrence.

## DISCUSSION

Primary SS is an immune-mediated exocrinopathy characterized by lymphocytic infiltration of the salivary and lacrimal glands, with insidious onset, increased frequency with age and a female predominance (9:1). Primary SS is frequently related to malignancies and especially non-Hodgkin lymphomas (NHLs). Patients with pSS are at higher risk of lymphoma than the healthy population (10- to 44-fold), usually mucosa-associated lymphoid tissue (MALT) lymphoma.^1^ The long-term risk of lymphoma for patients with pSS is estimated to be 5%. Mucosa-associated lymphoid tissue lymphoma may arise from different anatomical sites, including salivary and lacrimal glands, stomach, lungs and in the majority of cases it is a low differentiation marginal zone B cell lymphoma.^2^

Mucosa-associated lymphoid tissue lymphoma of the lung is reported to evolve in 20% of patients with pSS and usually may be restricted in lung parenchyma for long periods of time before being disseminated.^3^ Bronchus-associated lymphoid tissue lymphomas derive from bronchus-associated lymphoid tissue and account for less than 1% of all NHLs and 80% of all primary NHLs of the lungs.^4^ Bronchus-associated lymphoid tissue is absent from the lung under normal circumstances. Chronic antigenic stimulation in certain autoimmune disorders (sarcoidosis, systemic lupus erythematosus, rheumatoid arthritis, Hashimoto thyroiditis and particularly SS) are considered to influence the onset of BALT lymphoma. After years of slow evolution, these low-grade tumors may progress to rapidly growing high-grade lymphomas; features that may contribute to the differential diagnosis from other lung-involving lymphomas.^5^

Most patients are asymptomatic and pulmonary lesions are incidentally detected on a chest radiograph.^6^ Furthermore, some patients complain of dry cough, dyspnea and chest pain. Less than a quarter of patients have B-symptoms such as fever, night sweats, loss of body weight and pruritus. On chest auscultation, crackles are present in about 1/3 of patients.

Various studies have identified several risk factors for lymphoma development in patients with pSS, compared to the general population, that increase the overall mortality. Clinical features at the disease presentation, such as persistent salivary gland enlargement, lymphadenopathy, palpable purpura, peripheral neuropathy, the Raynaud phenomenon, serological features including RF, anti-Ro/SSA or/and anti-La/SSB autoantibodies positivity, monoclonal gammopathy, C4 hypocomplementemia, CD4 lymphocytopenia and cryoglobulinemia, as well as extensive lymphocytic infiltration in minor salivary gland biopsy (Tarpley score ≥3) were found to be associated with NHL development. Such patients should be monitored and managed closer than other patients with pSS.^7^ Ioannidis et al. found that the lymphoproliferative disease was independently predicted by parotid gland enlargement, palpable purpura and low C4 levels,^8^ while Tzioufas et al. proposed the presence of mixed monoclonal cryoglobulinemia as the most significant factor in predicting the risk of lymphoma development.^2^ Finally, elevated serum β2-microglobulin levels, the extinction of previously positive RF and long duration of SS (>7 years) are other biological predictors of NHL development in SS.^9^

In a recent case-control study of Fragkioudaki et al., a predictive model for NHL development in SS patients was identified. Investigators, based on initial clinical, laboratory and histopathological evaluation of SS patients, confirmed that salivary gland enlargement, lymphadenopathy, Raynaud phenomenon, anti-Ro, anti-La as well as RF positivity, monoclonal gammopathy and C4 hypocomplementemia are independent adverse predictors for NHL development. They developed a risk assessment tool, easily used in everyday practice, based on combinations of independent adverse predictors, allowing at the same time the designation of early preventive therapeutic strategies in high-risk SS patients for NHL development.^10^

Radiographically, there is a great variety of lesions regarding the pulmonary involvement of BALT lymphoma; such as nodules, solitary or multiple localized areas of consolidation with accompanying air bronchogram, peribronchial infiltrates, reticulation and bilateral ground glass opacities, especially at the lower lobes. The radiographical findings are non-specific and the most common are patchy bilateral infiltrates, irregularly and peripherally distributed in both lungs.^6^

Computed tomography imaging shows solitary or multiple nodules (<3cm in diameter) or masses (>3cm diameter), confluent alveolar opacifications with extensive peribronchial distribution with areas of air aerobronchogram, or ground glass attenuation. Typically, BALT lymphomas are located along a bronchovascular bundle and may be accompanied by traction bronchiectasis. Lesions usually involve the lower lobes. Interlobular septal thickening may also be present within or around the lesion.^6^ Very slow growth is characteristic of BALT lymphoma. High-resolution CT findings may resemble those of benign lymphoproliferative disorder. Involvement of mediastinal or hilar lymph nodes is associated with poor overall prognosis.^11^

The differential diagnosis of HRCT findings include disorders that spread along pulmonary lymphatics, such as bronchoalveolar adenocarcinoma, metastatic disease, sarcoidosis, lymphangitic carcinomatosis, cryptogenic organizing pneumonia or other malignant lymphoproliferative diseases.^11^ The absence of significant hilar and mediastinal lymphadenopathy, pleural effusions and areas of necrosis in conjunction with absence of significant symptoms are typical for patients with BALT lymphoma of the lungs, but not specific enough for definitive diagnosis. Flow cytometry analysis of bronchoalveolar lavage could be suggestive of the disease, but tissue documentation must be ensured.^2,6^

Diagnosis is definitely concluded through histopathologic examination.^4^ Tissue from the lungs can be obtained by transbronchial biopsy, CT-guided biopsy or open thoracotomy. Histopathology and immunostain tests are sufficient for diagnosis. Extensive infiltration of lung parenchyma by lymphoid proliferation, variable in intensity, comprised predominantly of small lymphocytes and plasma cells. Lesions show a denser pattern in the central region and more disperse or nodular infiltrate toward the periphery.^12^ The pattern of growth is interstitial, with prominent perivascular and peribronchial expansion.^11^ Airways and vessels of all sizes are involved. Lymphocytes infiltrate the lung in an intra-alveolar pattern, or by surrounding normal pulmonary structures, such as visceral pleura.^6^

Small parenchymal nodules may also be observed, resulting in the compression of small airways. In almost all cases of lung BALT lymphoma, reactive-appearing germinal centers around the infiltrate are observed. In the majority of cases the immunohistochemical studies are characterized by the expression of CD20 B cell phenotype and the absence of CD5, CD23, CD10 antigens as well as cyclin D1 proteins. Immunoglobulin light chain restriction is demonstrated and kappa or lambda light chain immunoglobulins are detected; especially monoclonal IgM-κ, IgM-λ and IgG-κ.^12^

Surgical excision, chemotherapy and radiation alone or in combination are the options for treatment of BALT lymphoma^13^ Different chemotherapeutic regimes are used successfully. The choice of therapy depends on the stage of the disease.^4^ Localized disease can be treated with surgery, radiation therapy or chemotherapy; while patients with disseminated disease require chemotherapy. The chemotherapeutic agents that are used are R-CVP (Rituximab, cyclophosphamide, vincristine and prednisolone), R-CHOP (Rituximab, cyclophosphamide, doxorubicin, vincristine and prednisolone) or FND (fludarabine, mitoxantrone and dexamethasone). However/chemotherapy should be considered as the first-line option in order to preserve lung function.^13^ Several studies have shown that both oxaliplatin as monotherapy and fludarabine are promising chemotherapeutic regimens. Rituximab is another option, added to classic chemotherapeutic agents, both as initial treatment or/and in cases of relapse. Overall response to treatment is satisfactory and prognosis is favorable.^6^

In conclusion BALT lymphoma of lung associated with pSS is characterized by discordance between clinical expression and radiological appearance. The wide spectrum of radiographic findings seems to reflect the equally wide spectrum of histopathological patterns of infiltration, and their possible combination during disease evolution. Clinical presentation and imaging findings are not specific enough to suggest MALT lymphoma of the lung; therefore histological confirmation is necessary for diagnosis. Various chemotherapeutic agents in combination with Rituximab lead to partial or complete remission in the majority of patients. The five-year survival rate exceeds 80%.
